# Uptake of purines in *Plasmodium falciparum*-infected human erythrocytes is mostly mediated by the human Equilibrative Nucleoside Transporter and the human Facilitative Nucleobase Transporter

**DOI:** 10.1186/1475-2875-9-36

**Published:** 2010-01-29

**Authors:** Neils B Quashie, Lisa C Ranford-Cartwright, Harry P de Koning

**Affiliations:** 1 Institute of Biomedical and Life Sciences, Division of Infection and Immunity, Glasgow Biomedical Research Centre, University of Glasgow, Glasgow G12 8TA, UK; 2 Centre for Tropical Clinical Pharmacology and Therapeutics, University of Ghana Medical School, PO Box GP 4236, Accra Ghana

## Abstract

**Background:**

*Plasmodium *parasites are unable to synthesize purines *de novo *and have to salvage them from the host. Due to this limitation in the parasite, purine transporters have been an area of focus in the search for anti-malarial drugs. Although the uptake of purines through the human equilibrative nucleoside transporter (hENT1), the human facilitative nucleobase transporter (hFNT1) and the parasite-induced new permeation pathway (NPP) has been studied, no information appears to exist on the relative contribution of these three transporters to the uptake of adenosine and hypoxanthine. Using the appropriate transporter inhibitors, the role of each of these salvage pathways to the overall purine transport in intraerythrocytic *Plasmodium falciparum *was systematically investigated.

**Methods:**

The transport of adenosine, hypoxanthine and adenine into uninfected and *P. falciparum*-infected human erythrocytes was investigated in the presence or absence of classical inhibitors of the hFNT1, hENT1 and NPP. The effective inhibition of the various transporters by the classical inhibitors was verified using appropriate known substrates. The ability of high concentration of unlabelled substrates to saturate these transporters was also studied.

**Results:**

Transport of exogenous purine into infected or uninfected erythrocytes occurred primarily through saturable transporters rather than through the NPP. Hypoxanthine and adenine appeared to enter erythrocytes mainly through the hFNT1 nucleobase transporter whereas adenosine entered predominantly through the hENT1 nucleoside transporter. The rate of purine uptake was approximately doubled in infected cells compared to uninfected erythrocytes. In addition, it was found that the rate of adenosine uptake was considerably higher than the rate of hypoxanthine uptake in infected human red blood cells (RBC). It was also demonstrated that furosemide inhibited the transport of purine bases through hFNT1.

**Conclusion:**

Collectively, the data obtained in this study clearly show that the endogenous host erythrocyte transporters hENT1 and hFNT1, rather than the NPP, are the major route of entry of purine into parasitized RBC. Inhibitors of hENT1 and hFNT1, as well as the NPP, should be considered in the development of anti-malarials targeted to purine transport.

## Background

Since purine salvage from the host milieu is an important physiological requirement for growth and multiplication of *Plasmodium falciparum*, purine transporters are regarded as ideal targets for the development of novel purine-based anti-malarial drugs to combat malaria [1-3].

Non-infected human erythrocytes take up nucleosides through the human equilibrative nucleoside transporter hENT1 [4] and purine bases through the facilitative nucleobase transporter hFNT1 [5,6]. hENT1, but not hFNT1, is potently inhibited by 6-[(nitrobenzyl)-thio]9-β-d-ribofuranosylpurine (NBMPR) [7]. Yet, while NBMPR completely blocks adenosine uptake in human erythrocytes, infection with *P. falciparum *induced an additional, NBMPR-insensitive, uptake mechanism in these cells [2,8] and this mechanism does not distinguish between the d and l-enantiomers of adenosine [9].

These observations helped define the concept of the new permeation pathway (NPP), an apparently non-saturable channel-like system that transports low molecular weight compounds including purines, and is formed after parasite invasion of erythrocytes [10]. Several researchers have described the NPP as exhibiting functional characteristics of an anion channel; being selective for Cl^- ^over K^+ ^and blocked by a range of classical anion channel inhibitors including furosemide and 5-nitro-2-(3-phenylpropylamino) benzoic acid (NPPB) [11-13]. Using the patch-clamp technique, two groups confirmed the presence and properties of the NPP in *P. falciparum*-infected erythrocytes [14,15].

While it is clearly established that the NPP is capable of mediating adenosine uptake, it is less clear whether this function is important in terms of its contribution to the overall purine salvage given the continued presence of both hENT1 and hFNT1 in parasitized RBC. It is also unknown whether hypoxanthine, the preferred purine of *P. falciparum *[16,17], is a permeant of the NPP. In the current study we thus investigated the degree to which the overall purine salvage by intraerythrocytic *P. falciparum *is dependent on the NPP. The transport of adenosine, hypoxanthine and adenine into *P. falciparum*-infected and uninfected human erythrocytes was, therefore, studied in the presence of selective inhibitors. The results show that despite the presence of the NPP, transport of nucleoside and nucleobase into infected RBC is largely through hENT1 and hFNT1, respectively.

## Methods

Transport assays with infected cells were performed with the standard 3D7 drug-sensitive laboratory clone of *P. falciparum*, originally obtained from David Walliker (School of Biological Sciences, University of Edinburgh, Edinburgh, Scotland, UK). Human blood and serum used for *Plasmodium *culture were obtained from the Glasgow and West of Scotland Blood Transfusion Service. Asexual parasites of *P. falciparum *were maintained in continuous culture using slightly modified standard methods [18]. Briefly, parasites were grown in RPMI 1640 medium supplemented with 5.94 g/l HEPES, 0.21% NaHCO_3 _and 10% heat-inactivated normal human serum at 5% haematocrit. The culture was incubated at 37°C under a gas mixture of 1% O_2_, 3% CO_2 _and 96% N_2_, and medium was changed daily. Prior to use in experiments, parasite cultures were synchronized using a previously described method [19].

Using the method described by the group of Kramer [20], synchronized cultures of parasitized erythrocytes at trophozoite stage were enriched for infected cells on isotonic Percoll™ (Amersham Biosciences) gradients (density 1.090 and osmolality 320 mosm (kg H_2_O)^-1^), resulting in a suspension with >80% parasitaemia. The trophozoite-enriched red blood cells harvested were washed three times with Erythrocyte Medium (140 mM NaCl, 5 mM KCl, 20 mM Tris/HCl (pH 7.4), 2 mM MgCl_2_, and 0.1 mM EDTA) and re-suspended in the same media for all the experiments performed in this study. In experiments that compared uptake into uninfected erythrocytes with parasitized erythrocytes, erythrocytes from the same donor were incubated in parallel with the infected erythrocyte cultures under the same conditions and were similarly centrifuged through Percoll before being used in the assay. Cell concentrations were determined with an improved Neubauer counting chamber.

All experiments with *P. falciparum *parasites were carried out using synchronized and Percoll enriched trophozoite-infected human erythrocytes. [2,8-^3^H]-Adenine (27 Ci/mmol) used in this study was purchased from Moravek (USA) whilst [2,8-^3^H]-adenosine (23 Ci/mmol) and [8-^3^H]-hypoxanthine (19 Ci/mmol) were obtained from Amersham Pharmacia Biotech (UK). Unlabelled adenine, hypoxanthine, adenosine and uridine of highest purity were obtained from Sigma. Transport of [^3^H]-adenosine, [^3^H]-adenine and [^3^H]-hypoxanthine into *P. falciparum*-infected erythrocytes and uninfected erythrocytes in the presence of NBMPR (Sigma), furosemide (Sigma), NPPB (Sigma) or 1 mM unlabelled permeant was determined exactly as previously described [21]. Briefly, equal volumes of a suspension of the cells (usually at a concentration of 4 × 10^7 ^cells/assay) and a radiolabeled permeant at twice its final concentration were mixed for predetermined times. Influx of extracellular radiolabeled permeant into the cells was terminated by the addition of 1 ml ice-cold stop solution followed by a quick spin at 13,000 × g to sediment cells through an oil-mix (300 μl of 5 parts dibutylphthalate (Aldrich): 4 parts dioctyl phthalate (Aldrich), v/v). The stop solutions used in the experiments in this study contain dilazep, furosemide, or papaverine (all from Sigma), to	 terminate uptake through hENT1, NPP and hFNT1, respectively. Cold stop solutions are designed to stop further uptake of permeant into the cells while the cells pellet below the oil-mix [5]. All experiments described in this study were performed in triplicate.

After the uptake of the permeant, the pellets were retrieved and processed for scintillation counting of radiolabel incorporated using the method previously described by Saliba and colleagues [22]. All uptake data presented in this study are presumed to represent a "mediated uptake," defined as the total uptake of the permeant apart from simple diffusion through the plasma membrane, which is not a saturable process. Non-mediated uptake of the respective permeant was assessed by determining the rate of uptake of the radiolabeled permeant in the presence of 1 mM unlabelled permeant as well as with cells and permeant at 0°C and subtracted from total uptake. The rate of uptake of the permeant was obtained from a plot of permeant concentration against time using the Prism 4 (GraphPad) software package. K_i _values were calculated from the equation K_i _= IC_50_/(1+ [L/K_m_]), where L is the permeant concentration. The K_m _and V_max _values were obtained for the transporters using the Michaelis-Menten plot.

## Results

**Figure 1:**
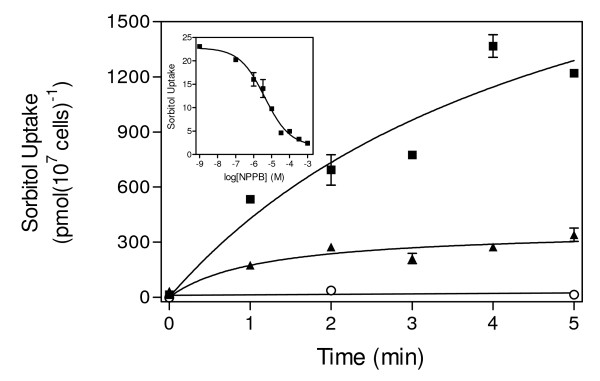
**Sorbitol transport in *Plasmodium falciparum*-infected and uninfected human erythrocytes in the presence or absence of NPPB**. Uptake of 10 mM [^3^H]-sorbitol into uninfected erythrocytes (open circles) and into Percoll-enriched *Plasmodium falciparum*-infected erythrocytes in the presence (solid triangles) or absence (solid squares) of 300 μM NPPB was determined over a period of five minutes as indicated. *Inset*: Inhibition of 10 mM [^3^H]-sorbitol (expressed as pmol(10^7 ^cells)^-1^s^-1^) into Percoll-enriched *P. falciparum*-infected erythrocytes by various concentrations of NPPB as indicated. All data shown are the average of triplicate determinations. Error bars represent SEM; where not shown, bars fall within the symbol.

Classical inhibitors such as the NPPB, NBMPR and furosemide were chosen for use in this study based on their published properties and subsequent verification in preliminary experiments in this study. We tested the effect of NPPB on the uptake of sorbitol, a well-known substrate of the NPP [11,23], into infected RBC. The presence of 300 μM NPPB inhibited [^3^H]-sorbitol uptake by over 95% in infected RBC; uninfected RBC, lacking the NPP, displayed no detectable [^3^H]-sorbitol uptake (Figure 1). These findings confirm the inhibitory effect of NPPB on the NPP [12] and are consistent with observations reported by Staines and colleagues [24]. A mean IC_50 _of 4.5 ± 1.8 μM (n = 3) was determined for the inhibition of sorbitol uptake by NPPB (Figure 1, insert).

### Nucleoside uptake

**Figure 2:**
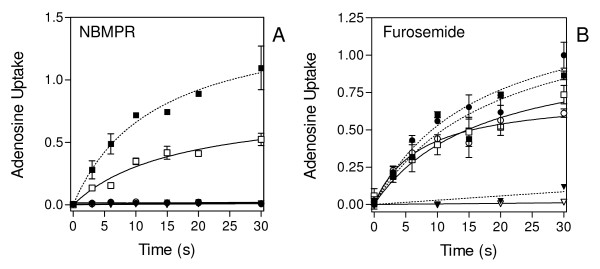
**Adenosine transport in *Plasmodium falciparum*-infected and uninfected human erythrocytes in the presence or absence of NBMPR**. Uptake of 0.5 μM [^3^H]-adenosine in Percoll-enriched infected (closed symbols, dashed lines) and uninfected (open symbols, solid lines) erythrocytes without inhibitor (squares), in the presence of inhibitor (circles) or 1 mM unlabelled adenosine (triangles). *A*, inhibitor is 0.1 μM NBMPR. Uptake in both infected and uninfected RBC was fully inhibited by either the presence of 1 mM adenosine or by 0.1 μM NBMPR. *B*, inhibitor is 100 μM furosemide. Adenosine uptake was not significantly reduced by 100 μM furosemide, either in infected or uninfected RBC. Uptake was expressed as pmol(10^7 ^cells)^-1^; experiments shown are representative of four independent experiments and conducted in triplicate; bars are SEM.

Uptake of adenine, hypoxanthine adenosine and uridine into *P. falciparum *infected or uninfected RBC in the presence or absence of NBMPR, furosemide or NPPB was studied. Uptake of [^3^H]-adenosine in *P. falciparum*-infected and uninfected human erythrocytes was linear (r^2 ^= 0.93 - 0.99) for at least 10 seconds, allowing the determination of uptake rates by linear regression over this interval. [^3^H]-Adenosine was rapidly taken up by uninfected human erythrocytes with a rate of 0.032 ± 0.006 pmol(10^7 ^cells)^-1^s^-1 ^with complete inhibition of uptake in the presence of 1 mM unlabelled permeant (Figure 2A, B). Uptake of 0.5 μM [^3^H]-adenosine was also 95% inhibited by 100 nM NBMPR (Figure 2A), consistent with the well-documented presence of the NBMPR-sensitive hENT1 adenosine transporter in human erythrocytes [4,7]. In contrast, adenosine uptake into human red blood cells (RBC) was not inhibited by 100 μM furosemide (Fig. 2B), an inhibitor of the *P. falciparum*-induced NPP [11]. Uptake of 0.5 μM [^3^H]-adenosine was faster (0.070 ± 0.005 pmol(10^7 ^cells)^-1^s^-1^) in *P. falciparum*-infected erythrocytes than in uninfected cells (0.033 ± 0.006 pmol(10^7 ^cells)^-1^s^-1^), and also reached a higher level (Figure 2), consistent with a generally higher level of nutrient uptake in infected cells as discussed by Kirk and colleagues [10]. This was not the result of significant levels of transport through the NPP, however, as adenosine uptake in infected cells was almost completely inhibited by NBMPR and was fully saturable (Figure 2). The rate of adenosine uptake in infected RBC was identical in the presence or absence of 100 μM furosemide (0.054 ± 0.006 and 0.059 ± 0.003 pmol(10^7 ^cells)^-1^s^-1^, respectively, for the experiment shown in Figure 2B).

**Figure 3:**
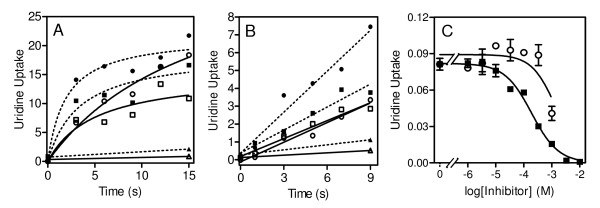
**Uridine transport in *Plasmodium falciparum*-infected and uninfected human erythrocytes in the presence or absence of NPPB**. *A*, *B *Uptake of 25 μM [^3^H]-uridine into *P. falciparum*-infected erythrocytes (closed symbols and dotted lines) and uninfected erythrocytes (open symbols, solid lines), expressed as pmol(10^7 ^cells)^-1^, was determined for the indicated times, at 22°C (*A*), or 8°C (*B*), respectively. Squares, no inhibitor; circles, 300 μM NPPB; triangles, 1 mM unlabelled uridine. Lines were calculated by linear regression and the results are the average of duplicate determinations. At 8 pmol(10^7 ^cells)^-1^, the intracellular concentration of uridine is estimated at 10.8 μM, using a published volume of 75 fL per infected erythrocyte [22]; thus, uridine has not reached equilibrium and linear rates of uptake are being measured over the interval of 9 s. *C*, Transport of 25 μM [^3^H]-uridine, expressed as pmol(10^7 ^cells)^-1^s^-1^, in uninfected human erythrocytes in the presence of various concentrations of NPPB (open circles) or unlabelled uridine (solid squares). Incubation time was 3 s and the experiment shown is representative of four independent experiments, each conducted in triplicate.

To demonstrate that the observation made with adenosine is genuine and is not attributable to purine metabolism within the cell, we repeated this assay using [^3^H]-uridine in the presence or absence of the NPP inhibitor NPPB; uridine is not metabolized by human erythrocytes or by *P. falciparum *[25-27]. At room temperature, uptake of 25 μM [^3^H]-uridine into the cells was too rapid to allow for accurate uptake rate measurements (Figure 3A) and the experiment was therefore repeated at 8°C (Figure 3B). At this temperature, uridine uptake into uninfected and infected erythrocytes in the absence of NPPB was estimated as 0.33 ± 0.03 pmol(10^7 ^cells)^-1^s^-1 ^and 0.43 ± 0.05 pmol(10^7 ^cells)^-1^s^-1^, respectively, showing an increase in infected cells as observed for adenosine uptake (see above), though the increase was less pronounced in the case of uridine. In uninfected cells, 300 μM NPPB did not inhibit the rate of uridine uptake (0.37 ± 0.03 pmol(10^7 ^cells)^-1^s^-1^), as expected for uptake through hENT1. Unexpectedly, NPPB appeared to increase uridine uptake in *P. falciparum*-infected erythrocytes, displaying a rate of 0.76 ± 0.08 pmol(10^7 ^cells)^-1^s^-1^. This difference was small but consistently observed both at 8°C and room temperature and sometimes also in the uninfected RBC. While no explanation immediately offers itself for this observation, it certainly does not indicate a significant contribution of uridine uptake by the NPP, a conclusion much strengthened by the near-complete inhibition by 1 mM unlabelled uridine (Figure 3A-C). Uridine uptake by hENT1 displayed a K_m _value of 177 ± 30 μM in uninfected RBC (n = 3; data not shown), consistent with the published value of 190 ± 12 μM [28], explaining the residual uptake at 1 mM unlabelled uridine. At 300 μM, NPPB did not affect hENT1-mediated [^3^H]-uridine uptake, but it inhibited this process by 20 - 50% at 1 mM (n = 3) (Figure 3C).

### Nucleobase uptake

**Figure 4:**
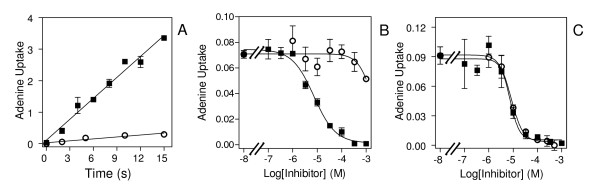
**Adenine transport in uninfected human erythrocyte**. *A*, Uptake of 0.25 μM [^3^H]-adenine into uninfected human erythrocytes in the presence (open circles) or absence (solid squares) of 1 mM unlabelled adenine, expressed as pmol(10^7 ^cells)^-1^. Experiment shown is representative of four independent experiments conducted in triplicate. *B *and *C*, Uptake of 1 μM [^3^H]-adenine, expressed as pmol(10^7 ^cells)^-1^s^-1^, by uninfected human erythrocytes over 5 s, in the presence or absence of various concentrations of unlabelled adenine (solid squares), NPPB (Frame *B*, open circles) or furosemide (Frame *C*, open circles). Experiments shown are representative of four independent experiments and conducted in triplicate. Bars represent SEM.

Uptake of 0.25 μM [^3^H]-adenine into uninfected RBC was found to be linear for up to 15 sec (r^2 ^= 0.98) with a rate of 0.22 ± 0.01 pmol(10^7 ^cells)^-1^s^-1^and was 93% inhibited by 1 mM unlabelled adenine (Figure 4A), in good agreement with previously published results [21]. The K_m _for [^3^H]-adenine in uninfected human erythrocytes was determined as 19 ± 1 μM (n = 3; data not shown), similar to the values of 13 ± 1 μM [5] and 16 ± 4.5 [21] reported earlier.

**Figure 5:**
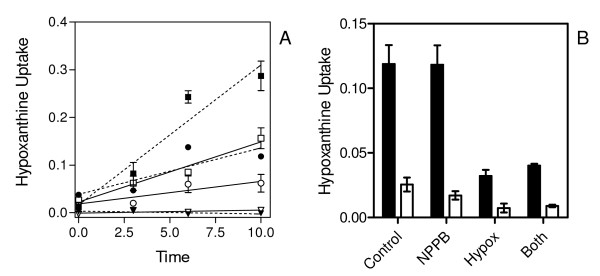
**Hypoxanthine uptake in uninfected and *P. falciparum*-infected human erythrocytes**. *A*, Uptake of 1 μM [^3^H]-hypoxanthine into *P. falciparum*-infected erythrocytes (closed symbols and dotted lines) and uninfected erythrocytes (open symbols, solid lines), expressed as pmol(10^7 ^cells)^-1^, was determined for up to 10 s: squares, no inhibitor; circles, 250 μM furosemide; triangles, 1 mM unlabelled hypoxanthine. Lines were calculated by linear regression and the results are the average of triplicate determinations. *B*, Bargraph of uptake of 0.5 μM [^3^H]-hypoxanthine in *P. falciparum*-infected human erythrocytes over a 2 s interval, expressed as pmol(10^7 ^cells)^-1^s^-1^. Bars are: Control, no inhibitor added; NPPB. 300 μM NPPB; Hypox, 1 mM hypoxanthine added; Both, 300 μM NPPB plus 1 mM hypoxanthine added; Closed bars and open bars represent identical experiments with two different batches of human erythrocytes, from different donors. Bars are mean and SEM of triplicate determinations at room temperature.

The hFNT1 nucleobase transporter is the only route of uptake for adenine and hypoxanthine in human erythrocytes [6,21] and it was next investigated whether this transporter is inhibited by inhibitors of the NPP. Like hENT1-mediated [^3^H]-uridine uptake, hFNT1-mediated [^3^H]-adenine uptake was not inhibited by NPPB at up to 1 mM (n = 3; Figure 4B). However, furosemide clearly inhibited hFNT1 with a K_i _value of 9.1 ± 1.7 μM (n = 4; Figure 4C). This was surprising as furosemide is not known to inhibit mammalian purine transporters. Thus, whilst uptake of 1 μM [^3^H]-hypoxanthine by infected RBC over the initial 10 s was 67% inhibited by 250 μM furosemide, uptake in uninfected RBC conducted in parallel was similarly inhibited (63%) (Figure 5A), providing scant evidence for the involvement of NPP in hypoxanthine uptake. Moreover, NPPB did not inhibit [^3^H]-hypoxanthine uptake in *P. falciparum*-infected human erythrocytes, and the residual [^3^H]-hypoxanthine uptake in the presence of 1 mM unlabelled hypoxanthine was not inhibited by the further addition of 300 μM NPPB either (Figure 5B). It was observed, however, that the rate of hypoxanthine uptake in infected RBC differed substantially from batch to batch, obtained from different donors. In some experiments, there did appear to be a level of inhibition by 300 μM NPPB, but the level of inhibition by NPPB plus 1 mM hypoxanthine was never more than with 1 mM hypoxanthine alone (Figure 5B, open bars) and overall it must be concluded from these experiments that the NPP contribution to hypoxanthine uptake is minor and not essential for parasite survival.

Finally, it was tested whether adenine competitively inhibits [^3^H]-hypoxanthine in infected RBC. In both uninfected and *P. falciparum*-infected erythrocytes, hypoxanthine uptake was completely blocked in the presence of 1 mM adenine. It is thus concluded that hypoxanthine, like adenosine, is overwhelmingly taken up through hFNT1 rather than NPP.

**Figure 6:**
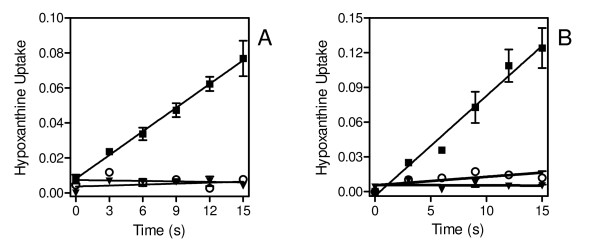
**Effect of adenine on hypoxanthine uptake into uninfected and *P. falciparum*-infected human erythrocytes**. Uptake of 0.5 μM [^3^H]-hypoxanthine, expressed as pmol(10^7 ^cells)^-1^, was determined in (*A*) uninfected and (*B*) *P. falciparum*-infected RBC at room temperature. Solid squares, no inhibitor control; open circles, 1 mM unlabelled hypoxanthine added; solid down triangles, 1 mM adenine added. The rate of uninhibited hypoxanthine uptake in infected and uninfected RBC was 0.0086 ± 0.0006 and 0.0045 ± 0.0001 pmol(10^7 ^cells)^-1^s^-1^, respectively; rates in the presence of 1 mM adenine and hypoxanthine were not significantly different from zero (P > 0.05; F-test). Experiments were performed in triplicate; error bars represent SEM.

Uptake of 1 μM [^3^H]-hypoxanthine into uninfected erythrocytes by the human facilitative nucleobase transporter (hFNT1) was generally 50-60% lower than in infected cells (see Figures 5A, 6A, B), consistent with the general upregulation of transport also noted with uridine and adenosine. Absolute rates varied with the batch of erythrocytes, thus experiments comparing rates between infected and uninfected erythrocytes were always performed in parallel, using the same batch of RBC, with uninfected treated exactly the same as the infected cells, including centrifugation through Percoll.

## Discussion

Purines, and in particular hypoxanthine, are essential nutrients that the intracellular malaria parasite requires for growth and multiplication [17,29]. Since parasites are only able to obtain these nutrients from the host milieu through salvage, availability of detailed information of the transporters involved in their uptake is crucial for the development of purine-based anti-malarial drugs. Much progress has been made in the last few years in identifying and characterizing purine transporters on the *P. falciparum *cell surface [1,30,31], and at least one of these transporters seems to be essential for growth at physiological purine concentrations [32].

The data presented in this manuscript clearly show that hypoxanthine enters *P. falciparum*-infected human erythrocytes overwhelmingly through a saturable, adenine-sensitive transport mechanism, which is compatible with the human FNT1 nucleobase transporter, but not with the properties reported for NPP [10,11]. Adenosine similarly enters predominantly through a saturable transporter, consistent with hENT1. The presence of these transporters on the plasma membrane of non-infected RBC has previously been reported [4]. Given the complexity of nutrient transport in the human erythrocyte, especially after *Plasmodium *invasion, it is imperative that a systematic approach be employed in order to obtain a genuine conclusion. Therefore, using classical transport inhibitors such as NPPB, furosemide and NBMPR, non-metabolized substrates such as uridine, and competitive inhibitors such as adenine for hypoxanthine uptake, the various pathways involved in purine uptake into *P. falciparum*-infected RBC were systematically dissected.

Prior to their use, the selectivity and IC_50 _values of these inhibitors was re-assessed to confirm which transporters are blocked and at what concentration. Uptake of sorbitol, a well-known substrate of the NPP [11,23], into infected RBC was almost fully blocked by NPPB. This uptake could not have occurred through any of the endogenous transporters, as there was no sorbitol uptake in uninfected erythrocytes. Similarly, it was verified whether known NPP inhibitors affected transport through the endogenous nucleoside and nucleobase transporters. NPPB did not affect either carrier, whereas furosemide was a good inhibitor of hFNT1, but not of hENT1. Although the mechanism for the furosemide effect on hFNT1 was not further investigated, the finding is potentially important, particularly towards efforts in search of purine-based antiplasmodial compounds, as similar concentrations of furosemide would inhibit both the NPP and hFNT1. The furosemide inhibition of [^3^H]-hypoxanthine and [^3^H]-adenine uptake reported here was clearly not through inhibition of the NPP as it was equally observed in infected and uninfected RBC. In view of the above inhibitor profile, it is evident that purine uptake in infected erythrocytes, being saturable and NPPB-insensitive, is mostly mediated by the human transporters hENT1 and hFNT1.

There is no doubt that nucleosides and nucleobases can enter the infected RBC through the furosemide/NPPB-sensitive new permeation pathways (NPP). For example, Gero and co-workers showed residual uptake of d-adenosine in infected human erythrocytes in the presence of the hENT1 inhibitor NBMPR [7], estimated at 20% of the uninhibited uptake rate [8], although this was not observed in the present study (Figure 2A). Kirk and colleagues [10] similarly demonstrated the NBMPR-insensitive, but partly furosemide and NPPB-sensitive, influx of adenosine and thymidine. However, that study used a concentration of 1 mM extracellular nucleoside, whereas the present study used a more physiological concentration of 0.5 μM. Whilst the higher concentrations were useful to demonstrate that nucleoside uptake through NPP is possible, the present investigation has sought to address the question whether the intracellular parasite is dependent on the NPP for purine uptake at physiological concentrations, which range from 0.1 - 1 μM for both adenosine and hypoxanthine [33,34]. Hypoxanthine uptake in *P. falciparum*-infected RBC was recently demonstrated, at 150 μM, to be ~50% inhibited by dantrolene, a newly discovered inhibitor of NPP, but the effect of dantrolene on hFNT1-mediated transport was not tested [35]. As it is here reported that furosemide also inhibits both the hFNT1 and NPP transport pathways, the possibility of similar action by dantrolene should not be discounted.

Notwithstanding the above, some of the reports from the group of Gero reported the NBMPR-insensitive uptake of d-adenosine at 1 μM [8,36]. They further showed uptake of l-adenosine and l-thymidine by *P. falciparum*-infected human erythrocytes, although these non-physiological enantiomers were not substrates for hENT1 [9,36]. Instead, uptake of l-nucleosides uptake was non-saturable and sensitive to furosemide [9] and NPPB [36], indicative of NPP. The potential of the NPP to take up low levels of purines, perhaps indiscriminately, may be of pharmacological importance, allowing the entry of toxic enantiomers and analogues selectively into the infected erythrocytes only.

Gero and colleagues also observed [2] that nucleoside transport inhibitors, including NBMPR, dilazep and dipyridamole all display intrinsic activity against *P. falciparum in vitro*, at concentrations that inhibit hENT1 [7]. This appears to show that inhibition of hENT1 leads to purine starvation of the developing parasite, which would be consistent with the observation that the rate of adenosine uptake is considerably higher than the rate of hypoxanthine uptake in infected RBC. Hypoxanthine is the preferred purine source for *P. falciparum *[17,29], but since adenosine is rapidly converted to hypoxanthine in the cytosol of the infected erythrocyte [37], it may be that inhibitors of hENT1 should have at least a growth-delaying effect on *P. falciparum in vivo *and that a combination with an inhibitor of hFNT1 would be lethal. It must be emphasized, however, that such a strategy would also deprive the uninfected erythrocytes of a purine source and that, while erythrocytes do not need purines for nucleic acid synthesis, their energy balance would be affected. However, it is likely that infected erythrocytes will be far more severely affected as their intracellular purine stores would be rapidly depleted by the highly efficient *P. falciparum *purine salvage system [38]. Yet, it is not certain that the above hENT1 inhibitors perform their anti-malarial activity by blocking the human adenosine transporter. For instance, Carter *et al *[31] report that the main *P. falciparum *nucleoside transporter, PfNT1, is 85% inhibited by 10 μM dipyridamole, although Parker *et al *[30] in a similar study did not find PfNT1 sensitive to dipyridamole. Furthermore, Gero and colleagues present clear evidence that NBMPR, in particular, is internalized and metabolized by the parasites [8]. Thus, while not transported by hENT1, the transport inhibitors could enter the Plasmodium-infected cells through NPP, and either inhibit the parasite's own purine transporters on its plasma membrane, or attack an intracellular target.

The data presented in the current manuscript show that uptake of adenosine in infected erythrocytes was almost completely inhibited by NBMPR and fully saturable - features which are inconsistent with the properties of the NPP. It may therefore be concluded that the NPP plays only a minor role in the salvage of this nucleoside in infected RBC. The observation that the rate of adenosine uptake in infected RBC was identical in the presence or absence of 100 μM of the NPP-inhibitor furosemide further supports this conclusion. Owing to the high rate of adenosine transport in erythrocytes, the equilibrative nature of hENT1, and the rapid metabolism of adenosine inside both infected and non-infected RBC, it is extremely difficult to measure true initial rates of [^3^H]-adenosine transport accurately in this system [7], and the data presented here refer to uptake, being the sum of transport and metabolism, rather than transport. However, the authors believe the conclusion that influx of adenosine is mediated by hENT1 rather than NPP is completely justified. To further validate these observations, the experiment was repeated using uridine as permeant, which is a substrate of hENT1, but not metabolized by human erythrocytes [26] or salvaged by *P. falciparum *[27]. The results show that, like adenosine, transport of uridine (25 μM) is overwhelmingly mediated by hENT1, being saturable and not inhibitable by NPPB.

This study clearly demonstrated that uptake of the nucleobases in infected human erythrocytes is also mainly through the exogenous hFNT1 transporter despite the presence of the NPP. As reported by Domin and colleagues, the non-infected human erythrocyte readily takes up adenine and hypoxanthine by facilitated diffusion through hFNT1 and, using ice-cold papaverine to instantly stop transport, it is possible to measure initial rates of transport over a brief but sufficiently long period [5]. In Figure 5A, the highest level of hypoxanthine uptake corresponds to an intracellular concentration of 0.39 μM, using the estimated volume of 75 fL for infected human erythrocytes reported by Saliba and co-workers [22], compared to an extracellular concentration of 1 μM and had thus not reached equilibrium. The uptake was clearly saturable and the response to NPPB was variable, resulting in a small increase in the uptake rate (Figure 5A) or, no difference, or a minor inhibition (Figure 5B). In addition, the study shows that initial rates of transport were completely inhibited by adenine (Figure 6), which cannot be explained either in terms of [^3^H]-hypoxanthine transport through NPP, or through effects on hypoxanthine metabolism: adenine does not intersect with hypoxanthine metabolism either in the erythrocyte or inside the parasite [37,39].

An interesting observation made in the current study is the apparent increase in the uptake rate of these nucleosides into infected RBC compared to uninfected erythrocytes. This was observed consistently for hypoxanthine, adenosine and uridine. While it is to be expected that infected RBC will take up more purines due to greater demand for nucleic acid synthesis from the intracellular parasite, these observations appear to reflect increased transport capacity, rather than increased usage. This is particularly obvious for uridine, which is not metabolized by Plasmodium. While this phenomenon has been reported previously for other nutrients, including tryptophan and choline (reviewed by Kirk and colleagues) [40], this had not yet been reported for purine transport across the infected erythrocyte membrane. The mechanism by which *Plasmodium *species increase nutrient uptake by host carriers is still a subject of intense debate.

## Conclusions

Taken together data presented in this study show that nucleobases and nucleosides present at low levels in the host plasma gain entry into the infected erythrocyte predominantly through equilibrative transporters rather than the parasite-induced New Permeation Pathways - a process efficiently driven by the rapid salvage of purines from the erythrocyte cytosol by the intracellular parasite, thus maintaining a concentration gradient across the RBC plasma membrane.

## List of abbreviations

hENT1: human Equilibrative Nucleoside Transporter 1; hFNT1: human Facilitative Nucleobase Transporter 1; NBMPR: 6-[(nitrobenzyl)-thio]9-B-d-ribofuranosylpurine; NPP: New Permeation Pathways; NPPB: 5-nitro-2-(3-phenylpropylamino) benzoic acid; PfNT1: Plasmodium falciparum Nucleoside Transporter 1; RBC: red blood cell.

## Competing interests

The authors declare that they have no competing interests.

## Authors' contributions

NBQ planned and carried out the research, performed preliminary analysis of the results, and drafted and revised the manuscript. LRC was involved in discussing the results and revised the MS. HdK planned the experiments together with NBQ, analysed the results, revised the manuscript and was responsible for overall strategy. All authors read and approved the final manuscript.
